# Forkhead box transcription factor 1 expression in gastric cancer: FOXM1 is a poor prognostic factor and mediates resistance to docetaxel

**DOI:** 10.1186/1479-5876-11-204

**Published:** 2013-09-03

**Authors:** Xiaoxiao Li, Wensheng Qiu, Bin Liu, Ruyong Yao, Shihai Liu, Yasai Yao, Jun Liang

**Affiliations:** 1 Department of Oncology, Affiliated Hospital of Medical College Qingdao University, 16 Jiangsu Road, Qingdao 266003, China; 2 Department of Nuclear Medicine, Affiliated Hospital of Medical College Qingdao University, Qingdao, China; 3 Central Laboratory, Affiliated Hospital of Medical College Qingdao University, Qingdao, China

**Keywords:** FOXM1, Gastric cancer, Prognosis, Docetaxel resistance

## Abstract

**Background:**

Forkhead box transcription factor 1 (FOXM1) has been reported to overexpress and correlate with pathogenesis in a variety of human malignancies. However, little research has been done to investigate its clinical significance in gastric cancer.

**Methods:**

We examined the expression of FOXM1 in 103 postoperational gastric cancer tissues and 5 gastric cell lines by immunohistochemistry and western blot analysis respectively. Data on clinic-pathological features and relevant prognostic factors in these patients were then analyzed. Moreover, the association of FOXM1 expression and chemosensitivity to docetaxel in gastric cancer cells was further explored.

**Results:**

Our study demonstrated that the level of FOXM1 expression was significantly higher in gastric cancer than in para-cancer tissues (P < 0.001) and normal gastric cell lines (P = 0.026). No significant association was found between FOXM1 expression and any clinical pathological features (P > 0.1). FOXM1 amplification was identified as an independent prognostic factor in gastric cancer (P = 0.001), and its affection is more significant in patients with tumor size larger than 5 cm (P = 0.004), pT3-4 (P = 0.003) or pIII-IV (P = 0.001). Additionally, shown to mediate docetaxel resistance in gastric cancers by our research, FOXM1 was revealed to alter microtubule dynamics in response to the treatment of docetaxel, and the drug resistance could be reversed with FOXM1 inhibitor thiostrepton treatment.

**Conclusions:**

FOXM1 can be a useful marker for predicting patients’ prognosis and monitoring docetaxel response, and might be a new therapeutic target in docetaxel resistant gastric cancer.

## Background

Despite the developments of surgical technique and improvements of anticancer drugs recently, gastric cancer is still the main cause of death and strongly associated with poor outcome. So far, in China, the morbidity of gastric cancer has reached to second with 3,621,000 new cases, whilst the mortality rate ranked third with the proportion of 14.33% each year [1]. Most patients present with metastatic or unresectable disease at the time of diagnosis [2]. For these surgically unfit but medically fit patients, palliative chemotherapy is the main choice of treatment. Among new generation chemotherapy regimens, docetaxel, which is a semisynthetic taxane, promoting the assembly and stabilization of microtubules to inhibit the depolymerization [3], has been used more and more extensively with potent effects [4-6]. The chemotherapy regimen of docetaxel, cisplatin and 5-fluorouracil (DCF) has been commonly used to treat the advanced stage or metastatic gastric carcinoma with encouraging survival outcomes [7-12] and better quality of life [13,14]. Yet, resistance to docetaxel does occur in gastric cancers [15]. Thus, identification of some suitable biomarkers for predicting patient prognosis and chemosensitivity is significant for improving therapeutic effects for patients with advanced gastric cancer.

Forkhead box protein M1 (FOXM1), characterised by a 100 amino acid winged-helix DNA binding domain, is a newly unified family member of Forkhead transcription factor [16]. Previous researches indicated that FOXM1, activated by the Ras-MAPK and hedgehog signaling pathway [17,18], played an important role in cell cycle by promoting both the transition from G1 to S phase and progression to mitosis through genes of Cdc25B, CDK1 and p27^KIP^ et al. [19,20]. It was demonstrated that FOXM1 overexpressed in gastric cancer and that elevated FOXM1 promoted tumor development in various kinds of cancers, correlated closely with poor outcome [21-23]. Additionally, in current research, FOXM1 amplification was reported to confer primary resistance of gefitinib in non-small cell lung cancer (NSCLC) and acquired paclitaxel resistance in breast cancer, showing implications in resistance to chemotherapy strongly [24,25]. These results suggested that FOXM1 may play an important role in progression of human cancers and may be associated with the resistance to docetaxel. Although FOXM1 has been revealed to mediate promotion of human gastric cancer angiogenesis, growth, and metastasis [26], the clinical significance of FOXM1 overexpression in gastric cancer is still little explored yet.

In current study, we examined the expression of FOXM1 protein in both gastric cancer specimens and cell lines, and assessed correlations among FOXM1 overexpression, clinic-pathological characteristics and clinic outcome. In addition, we investigated the relationship between overexpression of FOXM1 and docetaxel resistance in gastric cancer cells, trying to provide a support to its clinical significance in clinical practice.

## Materials and methods

### Human tissue specimens and patient information

Gastric cancer tissues were obtained from 103 patients who underwent gastrectomy and D2 lymphadenectomy at the Affiliated Hospital of Qingdao University Medical College from Jan 2007 to Nov 2007. 68 para-cancer tissues which were more than 5 cm away from the edge of tumor were randomly selected. All patients meet the criteria: 1) Tumors were confirmed to be gastric adenocarcinoma histologically. 2) None had received any preoperative treatment such as chemotherapy or radiotherapy. 3) Everyone was available of follow-up data. Clinic-pathological information was obtained from patient’s operative and pathological reports, in which age (≤50 years or >50 years), gender, size of tumor (≤5 cm or >5 cm), depth of tumor invasion (T1: tumor has invaded the mucosa or submucosa layer; T2: tumor has invaded the muscular layer; T3: tumor has invaded subserosa; T4: tumor invaded serosa or adjacent organs), lymph node metastasis, degree of differentiation (undifferentiated or differentiated),venous invasion, neural invasion, Borrmann type (Borrmann I, II, III, IV), Lauren type (intestinal, diffuse, mixed) and the 7th American Joint Committee on Cancer (AJCC) TNM stage (I, II, III, IV) were included.

### Cell lines and culture conditions

Three gastric cancer cell lines, SGC-7901, AGS, and MKN-28 and two normal gastric epithelium cell lines, GES-1 and HFE-145 were obtained from the central laboratory of Affiliated Hospital of Qingdao University Medical College. All these five cell lines were cultured in RPMI 1640 (Thermo scientific, CA), supplemented with 10% FBS (Thermo scientific, CA) and 1% Penicillin/Streptomycin (Invitrogen, Tokyo, Japan), and incubated in 5% CO_2_ at 37°C. The semisynthetic taxane, docetaxel (Selleckchem, US), was dissolved in dimethyl sulfoxide (DMSO) and diluted to a final concentration of 0.015 mg/L and 0.020 mg/L before use.

### Immunohistochemistry

The expression of FOXM1 was detected through immunohistochemistry (IHC) analyses with 3-um-thick sections of formalin-fixed and paraffin-embedded blocks. For IHC staining, tissue sections were dewaxed in xylene and rehydrated gradually with graded ethanol. For antigen retrieval, all the sections were incubated by microwave oven in citrate buffer solution (pH 6.0) for 20 minutes. Endogenous peroxidase was inactived by 0.3% hydrogen peroxide in methanol for 15 min. After that, tissue slides were incubated with rabbit polyclonal antibody against human FOXM1 (dilution 1:100, Epitomics, US) at 4°C overnight and then visualized antibody binding sites with the SP peroxidase detection system. Finally, sections were incubated in 3,3’-diaminobenzidine tetrahydrochloride for 3–10 minutes and restained with 0.1% hematoxylin. In every case, negative control reaction was set with PBS replacing FOXM1 antibody, while the known positive-stained section was used as positive control. The results of IHC were evaluated by two pathologists independently with no knowledge of clinic-pathological features.

The expression of FOXM1 was scored by multiplying the intensity scores and the percentage area positively stained [27,28]. Briefly, the intensity score was categorized into four groups: no staining marked 0; weak staining marked 1; moderate staining marked 2 and strong staining marked 3. For the percentage of positively stained cells, the scores were sorted from 0 to 4 (<5% marked 0; 5%-25% marked 1; 25%-50% marked 2; 51%-75% marked 3; 75% marked 4). After such calculation, the finally composite scores were divided into four grades: 0–1 were negative, 2–4 were weakly positive, 5–8 were moderately positive and 9–12 were strongly positive. 2 and more than 2 scores were evaluated as positive results.

### Western blot analysis

Whole-cell lysates were prepared from gastric cancer cell lines which were in logarithmic growth phase or at indicated periods of time. Total proteins were fractionated using sodium dodecyl sulfate polyacrylamide gel electrophoresis and transferred onto Polyvinylidene fluoride membrane. Anti-FOXM1 (dilution 1:1000, Epitomics, US) and anti-GAPDH (dilution 1:2000, CWBIO, CA) rabbit polyclonal antibodies were used as primary antibodies. For tubulin fractionation, α-tubulin antibody (1:1000, Santa, US) and β-tubulin antibody (1:1000, Santa, US) were used for analysis. The signals were detected using the VILBER enhanced chemiluminescence system (VILBER LOURMAT, FRA) according to the manufacturer’s instructions. Results shown were derived from at least three independent experiments.

### MTT assay

For MTT assays, 5000 cells were seeded overnight in 96-well plates and then cultured in 100 μL of fresh medium that contained various concentrations of docetaxel for 24–72 hours. Each concentration was repeated in triplicate. After that, MTT solution (20 μL, 5 mg/mL in PBS) was added to each well and the plate was incubated at 37°C for 4 hours. The solution was then removed and 200 μL of DMSO was added to each well. After 10 minutes of vibration mixing, the optical density (OD) at 490 nm was measured using a microplate reader (Bio RAD, US).

### Plasmids and transfections

The human FOXM1 expression vector pcDNA3.1-FOXM1 and siRNA-FOXM1 were obtained from the center library of Affiliated Hospital of Qingdao University Medical College. For transfections, cells were seeded to a 40–50% confluence state and transfected with pcDNA3.1-FOXM1, siRNA-FOXM1 or pcDNA3.1 with Lipofectamine 2000 agent (Invitrogen, US) in accordance with the manufacturer’s protocol. After transfection, cells were cultured for 48 h and analyzed by western blotting and MTT assay.

### Semi-quantitative RT-PCR

Total cellular RNA was extracted from cell pellets of each cell lines with Trizol reagent. The amount of RNA was determined by BJKO assay system (BJKO, CA), and part of them was reverse transcribed using the Reverse Transcription System (TaKaRa, CA). The primer sequences for PCR amplification were as follows: FOXM1 sense 5′- TAT TCA CAG CAT CAT CAC AGC A-3′ and antisense 5′- GAA GGC TCC TCA ACC TTA ACC T-3′; GAPDH sense 5′- ACC ACA GTC CTG CAT GCC AC -3′ and antisense 5′ - TCC ACC ACC CTG TTG CTG TA -3′. To ensure experiment accuracy, all reactions were performed in triplicate. The integrity of all RNA samples was verified by RT-PCR for GAPDH in each sample through gel imaging systerm (VILBER LOURMAT, FRA). The value of FOXM1 expression was divided by that of GAPDH in each sample.

### Molecular evolution assay

The gastric cancer cell line AGS was treated with 0.015 mg/L docetaxel for 72 hours when cells reached a confluency of 80%. After treatment, docetaxel containing medium was replaced by fresh medium. As soon as cells recovered, they were seeded for RNA isolation, cell lysis (protein), MTT assays and the next treatment cycle. In this manner, several rounds of molecular evolution assay were performed. To obtain the appropriate rate of cell death in the molecular evolution assay, several docetaxel concentrations were tested in a preliminary experiment. Thereby, 0.015 mg/L was obtained as the most suitable concentration.

### Tubulin assay

Separation of polymerized and soluble fractions was done in accordance with previously published assays [25]. Cells were seeded at 80% confluency in 24-well plates. The following day they were treated with 0 or 0.020 mg/L docetaxel for 48 hours. Cells were collected in hypotonic buffer (1 mM MgCl_2_, 2 mM EGTA, 0.5% Nonidet P-40, 20 mM Tris–HCl pH 6.8) and centrifuged for 10 minutes at room temperature (14,000 rpm). The supernatant was used as the soluble fraction while the pellet made up the polymerized fraction. Samples were analyzed by western blot.

### Follow-up

We performed a 5-year retrospective, cohort research for individual patients after gastric cancer surgery from 2007 to 2012. The median duration of follow-up was 49.2 months (range, 7–60 months). For patients who remained alive until the cut-off date, survival data were recorded as 60 months. The information about survival was collected in the Oncology Department of the Affiliated Hospital of Medical College Qingdao University. Also, informed consent was obtained from all the patients. The ethical committee of our institute approved the research protocol for this study.

### Statistical analysis

We used Chi-Square statistical test to identify correlations in the human gastric cancer clinic-pathological parameters and Log-rank analysis for the in vivo (patients) survival study. In addition, we evaluated significant differences in vitro data using Student’s t-test. A significance level set at p < 0.05 was considered significant for all the tests.

## Results

### FOXM1 protein expression in gastric cancer tissues and cell lines

**Figure 1:**
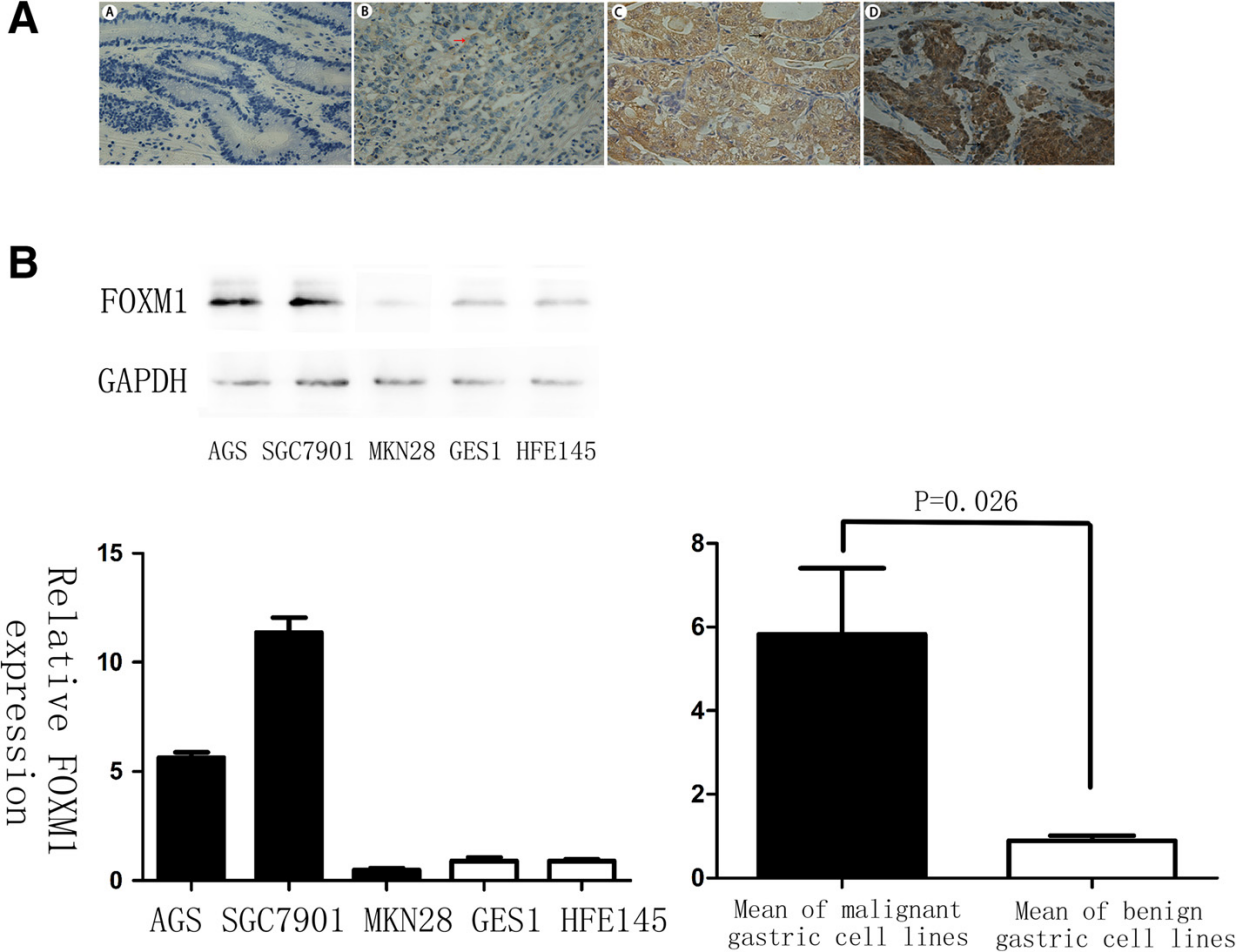
**FOXM1 expression in gastric cancer tissues and cell lines. (A)** Immunohistochemical staining for FOXM1 antibody in gastric cancer tissue: (A). Negative staining for FOXM1, ×400; (B). Weakly positive staining for FOXM1, ×400; (C). Moderately positive staining for FOXM1, ×400; (D). Strongly positive staining for FOXM1, ×400. FOXM1 expression was stained in the nuclei or cytoplasm of cells. Red arrow: cytoplasm stained; Black arrow: nuclei stained. **(B)** Different expressions of FOXM1 in gastric cancer cell lines: SGC-7901, AGS and MKN-28; normal gastric epithelium cell lines: GES-1 and HFE-145. There was a significant difference in FOXM1 expression between the benign and malignant gastric cell groups (P = 0.026).

FOXM1 was positively stained in the nuclei or cytoplasm of 79% gastric cancer cells, whereas the para-cancer tissues showed much lower levels of FOXM1 with nonspecific weak staining (11.8%) (Figure 1A). Among the 103 gastric cancer specimens, 12 cases were detected as strongly positive for FOXM1 expression (12%), 38 cases were moderately positive (37%), 31 cases were weakly positive (30%), and 22 cases were negative (21%) (Table 1). When further investigate the relationship between FOXM1 expression and clinic-pathlogical features in these gastric cancer patients, no significant association was found between the expression of FOXM1 and any clinic-pathological parameters, even no tendency can be seen (Table 2, P > 0.1). Overall, IHC results revealed that the level of FOXM1 expression was significantly higher in gastric cancer than in para-cancer tissues (Table 1, P < 0.001), indicating that FOXM1 was commonly overexpressed in human gastric cancers.

**Table 1:** Different FOXM1 expressions in gastric cancer tissues and paraneoplastic tissues

Variable^*^	**Gastric cancer**	**Paraneoplastic tissues**	**n**	**x**^**2**^	**P**
**Negative**	22	60	82	81.075	<0.001
**Weakly positive**	31	4	35		
**Moderately positive**	38	3	41		
**Strongly positive**	12	1	13		
**Entire group**	103	68	171		

Noted *: FOXM1 expression was graded according to Remmele and Halon [27,28] modified by authors.

**Table 2:** Association between FOXM1 expression and clinic-pathological factors in 103 patients after gastrectomy

**Variable**		**Entire group (n = 103)**	**FOXM1 negative**	**FOXM1 positive**	**P**
**Age**	≤50 years	17	4	13	1
	>50 years	86	18	68	
**Gender**	Male	68	15	53	0.809
	Female	35	7	28	
**Size**	≤5 cm	73	15	58	0.754
	>5 cm	30	7	23	
**Depth of tumor invasion**	T1-2	40	7	33	0.446
	T3-4	63	15	48	
**Lymph node metastasis**	Negative	40	8	32	0.789
	Positive	63	14	49	
**Degree of differentiation**	Undifferentiated	75	17	58	0.596
	Differentiated	28	5	23	
**Venous invasion**	Negative	61	14	47	0.701
	Positive	42	8	34	
**Neural invasion**	Negative	56	10	46	0.344
	Positive	47	12	35	
**Bomann histologic classifications (n = 77)**	BomannI	4	1	3	0.436
	BomannII-III	66	14	52	
	BomannIV	7	3	4	
**TNM staging**	I-II	45	7	38	0.206
	III-IV	58	15	43	
**Lauren type**	Intestinal	87	18	69	0.871
	Diffuse	13	3	10	
	Mixed	3	1	2	

Noted ^*^: p < 0.05.

To further confirm that results in vitro, we next investigated the level of FOXM1 expression in benign and malignant human gastric cell lines by western blot analysis. As a result, the gastric cancer cell lines equally exhibited 6-fold more FOXM1 expression compared with the normal ones (Figure 1B, P = 0.026), in which a homogenous FOXM1 expression was showed in the gastric epithelium cell lines such as GES-1 and HFE-145, whereas FOXM1 expression levels were more heterogeneous and in total elevated in cancerous cell lines, demonstrating that FOXM1 also overexpressed in human gastric cancer cell lines.

### Prognostic significance of FOXM1 expression in survival of gastric cancer patients

**Figure 2:**
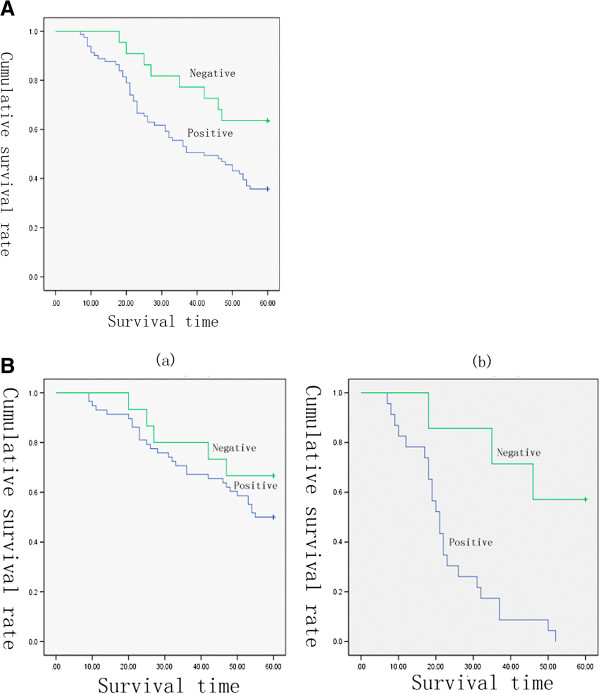
**Kaplan-Meier survival curves for postoperational gastric cancer patients. (A)** Kaplan-Meier survival curves in patients after gastrectomy according to FOXM1 expression (n = 103) (P =0.030). **(B)** Survival curves for the 103 gastric cancer patients with different tumor size according to FOXM1 expression. **(a)** Survival curves for the 73 patients with tumor size ≤ 5 cm according to FOXM1 expression (P = 0.294). **(b)** Survival curves for the 30 patients with size of tumor > 5 cm according to FOXM1 expression (P = 0.004).

The survival curves of patients after gastrectomy according to FOXM1 expression were showed in Figure 2A. The data demonstrated that 5-year survival rate was significantly lower in patients with positive FOXM1 expression than those without (P = 0.030). Univariate analysis revealed that tumor size, T stage, lymph node metastasis, vascular invasion, FOXM1 expression, and TNM stage were associated with an inferior survival duration (Table 3, P < 0.05). However, only the tumor size, T stage, FOXM1 expression and TNM stage were verified to be independent prognostic factors for the survival in gastric cancer patients after multivariate analysis (Table 3, P < 0.05).

**Table 3:** Results of univariate and multivariate analysis of 5-year survival by Cox proportional hazard model

**Variable**		**Univariate analysis**			**Multivariate analysis**		
	**n**	**OR**	**95.0% CI**	**P**	**OR**	**95.0% CI**	**P**
**Age (≤50 years / >50 years)**	19/84	0.738	0.363-1.499	0.4			
**Gender (F/M)**	68/35	0.759	0.440-1.308	0.32			
**Size of tumor (≤5 cm / >5 cm)**	73/30	0.287	0.170-0.483	0.001^**^	0.532	0.306-0.925	0.025^*^
**Depth of tumor invasion (T1-2/T3-4)**	40/63	0.209	0.108-0.404	0.002^**^	7.798	1.993-14.561	0.009^**^
**Lymph node metastatis (negative/positive)**	40/63	0.179	0.090-0.355	0.002^**^	0.457	0.192-1.086	0.076
**Degree of differentiation (undifferentiated/differentiated)**	75/28	1.041	0.593-1.825	0.889			
**Venous invasion (negative/positive)**	61/42	0.375	0.225-0.628	0.001^**^	0.758	0.433-1.326	0.332
**Neural invasion (negative/positive)**	56/47	0.917	0.553-1.523	0.739			
**FOXM1 expession (negative/positive)**	22/81	2.277	1.081-4.796	0.030^*^	6.251	2.699-14.478	0.001^**^
**TNM staging (I-II/III-IV)**	45/58	0.118	0.059-0.237	<0.001^**^	0.012	0.001-0.099	<0.001^**^
**Lauren type (intestinal/diffuse and mixed)**	87/16	0.700	0.318-1.541	0.376			

Noted ^*^: p < 0.05; ^**^: p < 0.01.

In order to explore the influence of different FOXM1 status on other independent prognostic factors, we performed a stage-stratified analysis of tumor size, pT, and pTNM according to FOXM1 expression levels. Figure 2B showed that when the tumor size is smaller than 5 cm, no obvious difference was observed between the survival duration in patients with different FOXM1 status. Conversely, when tumor size is larger, the five-year survival was significantly worse in those with positive FOXM1 expression than negative ones, indicating that FOXM1 expression significantly shortened the survival in patients with larger tumor size (Table 4, P = 0.004). Moreover, in the following analysis, the expression of FOXM1 was also found to significantly affect the survival in patients with stages T3-T4 (Table 4, P = 0.003) or stages III-IV (Table 4, P = 0.001), from which we can infer that at the same depth of tumor invasion or same stage, patients with FOXM1 amplifications could have a poorer survival prognosis than others (except for patients with stage T1-2 and I-II disease).

**Table 4:** Results of tumor size, pT, and pTNM stage-stratified analysis according to FOXM1 expression

**Variable**	**OR**	**95.0% CI**	**P**
**T1-2**	1.032	0.223-4.777	0.968
**T3-4**	3.758	1.589-8.886	0.003^*^
**≤5 cm**	1.662	0.643-4.294	0.294
**>5 cm**	6.206	1.784-21.59	0.004^*^
**I-II**	6.986	0.023-12.203	0.362
**III-IV**	3.901	1.800-8.457	0.001^*^

Noted ^*^: p < 0.01.

### FOXM1 expression levels of cell lines and sensitivity to docetaxel

**Figure 3:**
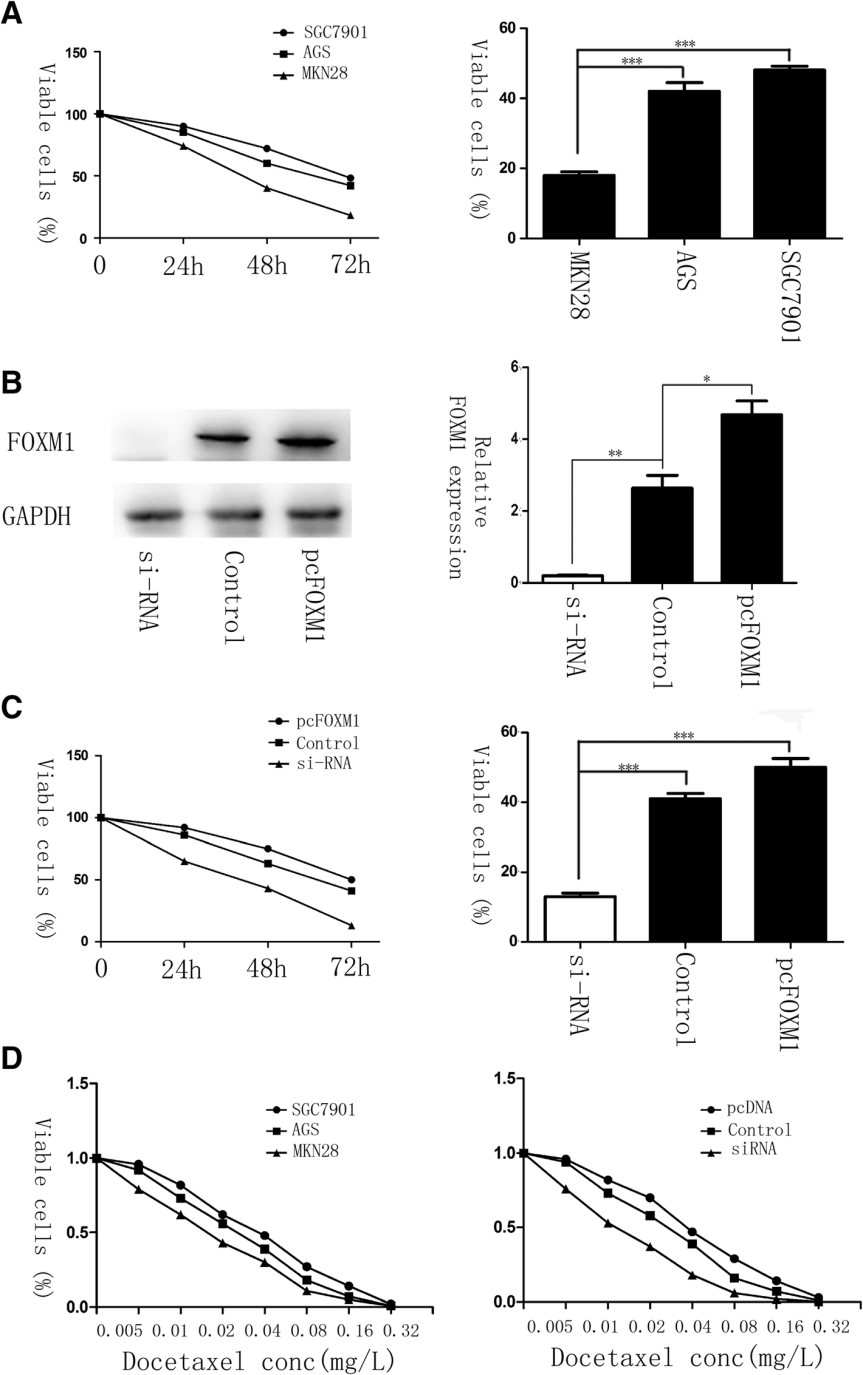
**Elevated levels of FOXM1 correlate with resistance to docetaxel in gastric cancer. (A)** AGS, SGC-7901 and MKN-28 cells were treated with 0.02 mg/L of docetaxel for 0, 24, 48 and 72 h. MTT assay was performed to test the cell viability. The IC50 calculations in the three cell lines were 0.034 mg/L, 0.025 mg/L and 0.016 mg/L separately **(D left)**. **(B)** The expression of FOXM1 in AGS cell lines with the transfection of FOXM1-siRNA, pcDNA3,1 or pcDNA3, 1-FOXM1, analyzed by western blot 48 h later. **(C)** Gastric cell lines AGS were treated with docetaxel at the concentration of 0.02 mg/L after FOXM1-siRNA or pcDNA3, 1-FOXM1 transfection. Cell growth curves were drawn by MTT assays. The IC50 in FOXM1 knockdown, overexpressed and control groups was 0.012 mg/L, 0.025 mg/L and 0.038 mg/L respectively **(D right)**. *, P ≤ 0.05; **, P ≤ 0.01; ***, P ≤ 0.001 significant.

In order to explore whether there is an association between the expression of FOXM1 and chemotherapy response to docetaxel, the human malignant gastric cell lines SGC-7901, AGS, and MKN-28, which has different expression levels of FOXM1, were treated with 0.02 mg/L of docetaxel for 72 hs. MTT assay was performed to test drug sensitivity. After treatment, only 15–20% of the MKN-28 cells survived, whereas the survival in FOXM1 overexpressed cells was greater than 40% (Figure 3A, P < 0.001). The IC50 of SGC-7901, AGS and MKN-28 cell lines was 0.034 mg/L, 0.025 mg/L and 0.016 mg/L respectively (Figure 3D left), indicating that the expression of FOXM1 correlated with docetaxel therapeutic efficacy significantly. To further confirm this result, we transfected pcDNA3.1-FOXM1 and FOXM1-siRNA into AGS cell lines (Figure 3B), and incubated them at the same drug concentration for 3 days. As shown by cell growth curve, the cell viability was absolutely lower in samples lacked FOXM1 expression, whilst the pcDNA3.1-FOXM1 transfected cells had higher viable rate (Figure 3C, P < 0.001). Moreover, the hypothesis that knockdown of FOXM1 in AGS sensitized the cells to docetaxel treatment was also evidenced by IC50 calculations, 0.038 mg/L (pcDNA3.1-FOXM1) and 0.025 mg/L (control) vs. 0.012 mg/L (siRNA FOXM1) (Figure 3D right). These data indicated that elevated FOXM1 can protect cells from docetaxol induced cell damage.

### Molecular evolution of gastric cancer cells lead to a docetaxel resistant phenotype and up-regulation of FOXM1

**Figure 4:**
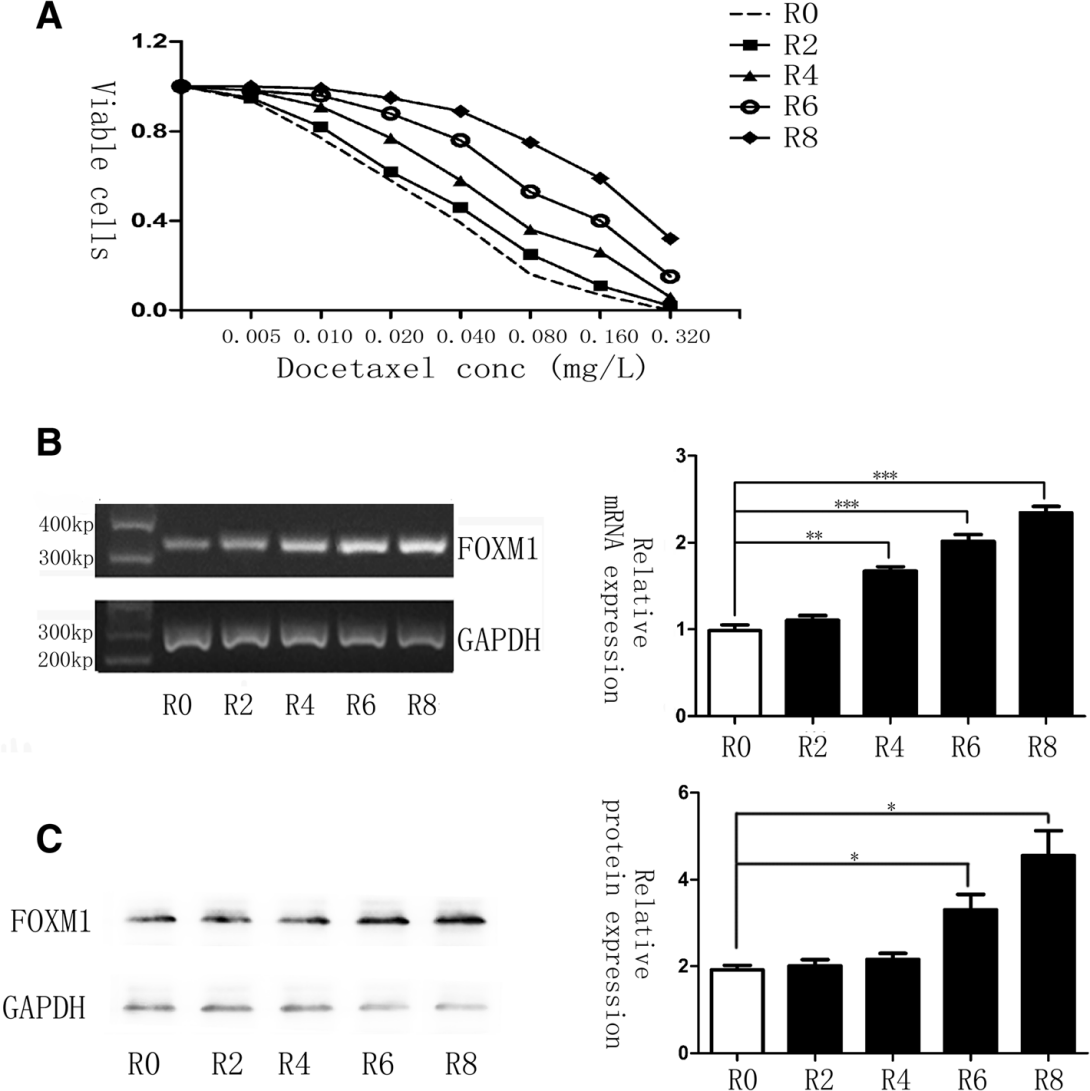
**Docetaxel resistant cell line shows elevated FOXM1 mRNA and protein expression levels. (A)** Cells in different cycle of molecular evolution assay were treated with docetaxel respectively, and their rates of cell viability were measured by MTT assay. R0 represents the untreated control cell line, whereas R2, R4, R6 and R8 represents AGS cells that are treated with 0.02 mg/L docetaxel for two, four, six and eight times, separately. IC50 for cells in R0, R2, R4, R6 and R8 was 0.026, 0.033, 0.054, 0.098, 0.190 mg/L. **(B)** FOXM1 mRNA transcript levels in R0, R2, R4, R6 and R8 cells were determined by RT-PCR analysis. **(C)** Western blot analysis was performed to detect the relative protein expression levels of FOXM1 in the different treated round. Statistical analysis was performed using Student’s t tests. *, P ≤ 0.05; **, P ≤ 0.01; ***, P ≤ 0.001 significant.

To confirm that chemoresistance can also lead to the up-regulation of FOXM1, we established the molecular evolution assay, where the malignant human gastric cell line AGS was treated with docetaxel for several cycles. After each treatment round, cells were harvested for MTT assays as well as RNA and protein isolation to investigate chemosensitivity changes and gene expressions. As a result, MTT assays revealed that cells in sequential treatment cycles had increasing IC50 calculations (Figure 4A), demonstrating that the resistance to docetaxel rose especially from the fourth treatment cycle on. In addition, changes in the levels of FOXM1 could be observed simultaneously. PCR result showed that the level of FOXM1 was significantly up-regulated after the fourth treated round (Figure 4B, P < 0.05), while the expression of FOXM1 altered correspondingly with mRNA levels (Figure 4C, P < 0.05). Based on such treatment cycle, AGS cells were finally succeed to have a good tolerance of docetaxel to the concentration of 0.2 mg/L, and were regarded as the AGS-DOC^R^ cell lines. These results provided another aspect of evidence and fully proved that FOXM1 could mediate the therapeutic resistance to docetaxel in gastric cancer.

### FOXM1 confers resistance to docetaxel by altering microtubule dynamics in preventing docetaxel induced apoptosis

Several mechanisms to combat palitaxol induced apoptosis have been reported previously. Namely, up-regulation of MDR1 (multi-drug resistant protein 1), a P-Glycoprotein family member can shuttle toxins out of cells; up-regulation of the CIAP (inhibitors of apoptosis) family members including Survivin; and the altered microtubule dynamics [29]. Given the overlapping roles of docetaxel and FOXM1 upon affecting the microtubule dynamics of mitosis progression in tumor cells, we suggested hypothesis that altered microtubule dynamics mediated by FOXM1 could prevent docetaxol induced apoptosis, which caused docetaxel resistance in gastric cancers.

**Figure 5:**
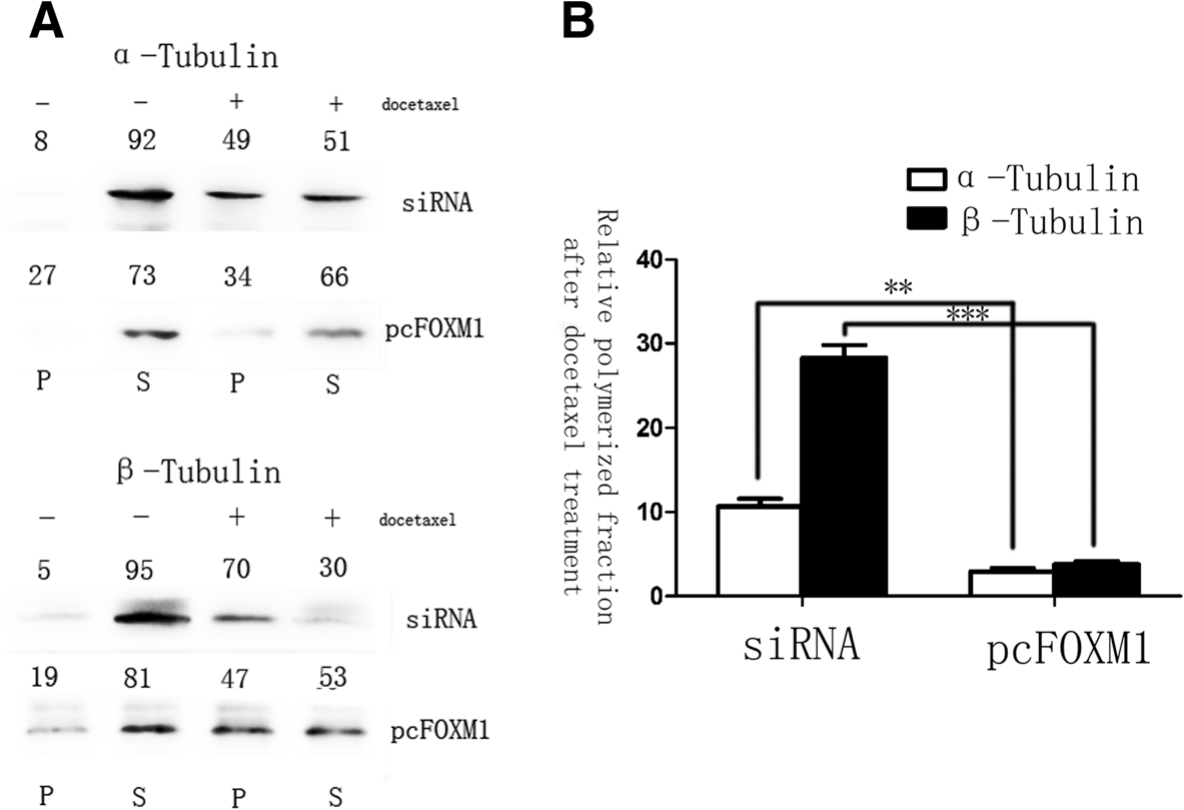
**FOXM1 alters the microtubule dynamics in preventing docetaxel induced apoptosis.** Polymerized and soluble tubulin fractions from docetaxel untreated and treated FOXM1-siRNA and pcDNA3, 1-FOXM1 transfected cell lines were generated by centrifugation. Western blot was used to assay α-tubulin and β-tubulin ratios in polymerized and soluble fractions. **(A)** Relative percentages are shown above western blot. **(B)** The soluble to polymerized microtubule fractions after docetaxel treatment were significantly inhibited in FOXM1 overexpressed group, analyzed by t test both for α-tubulin and β-tubulin. **, P ≤ 0.01; ***, P ≤ 0.001 significant.

To examine its possibility, we compared the ratio of soluble to polymerized microtubule fractions after docetaxel treatment. Cell lysates were fractionated to obtain polymerized and soluble tubulin fractions in FOXM1-siRNA transfected and FOXM1 overexpressed gastric cell lines that were left untreated or treated with docetaxol. Without drug treatment, cells showed similar tubulin ratios and most of the detectable tubulins were in the soluble form. Upon treatment with docetaxol, FOXM1 knockdown cells showed a dramatic shift towards the polymerized fraction. The FOXM1 expressing cells did show a shift towards the polymerized fraction but the ratio was significantly lower (Figure 5, P < 0.01). These data clearly indicated that through interfering in microtubule polymerization, the antitumor activity of docetaxel was inhibited by FOXM1 overexpression in gastric cancer cells, actually testifying our previous hypothesis.

### Thiostrepton can overcome docetaxel resistance in gastric cancer cells through down-regulation of FOXM1

**Figure 6:**
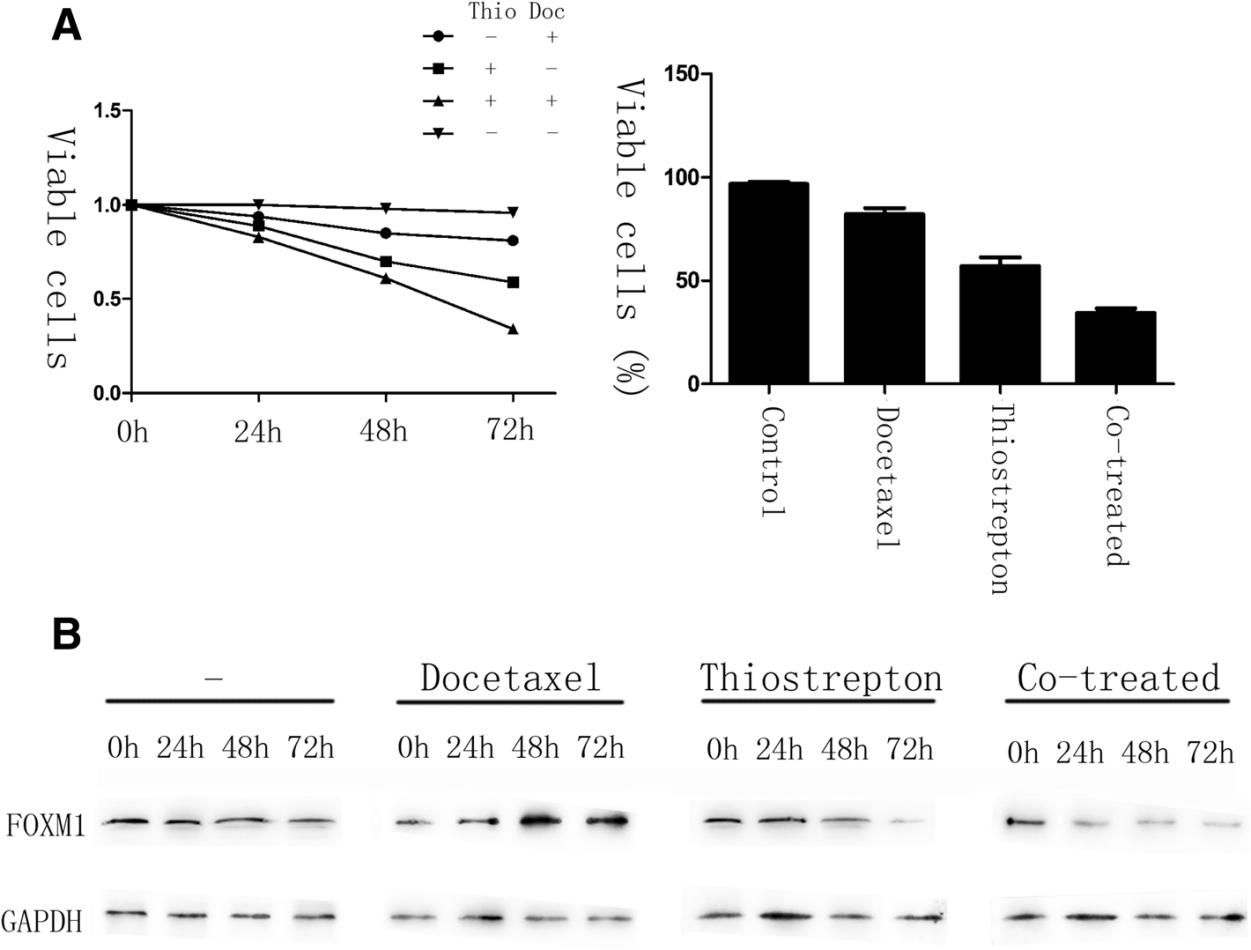
**Thiostrepton can reverse docetaxel resistance in gastric cancer cells.** AGS-DOC^R^ cells were treated with DMSO (vehicle control), 0.02 mg/L docetaxel, 16 mg/L thiostrepton or a combination of 0.02 mg/L docetaxel and 16 mg/L thiostrepton for 72 h. **(A)** The percentage of viable cells in different treatment group is shown at each time point by MTT assay. **(B)** Cell lysates were prepared at 0, 24, 48, and 72 h after treatment, and the expression of FOXM1 and GAPDH were analyzed by western blot analysis.

To test if FOXM1 inactivation is a viable strategy for overcoming docetaxel resistance, we studied the effects of AGS-DOC^R^ cells treated with FOXM1 inhibitor thiostrepton [30]. In result, MTT assays revealed that docetaxel resistant cells exhibited a significant reduction in the rate of cell viability after treated with thiostrepton or in combination with docetaxel compared with the single treatment of docetaxel. 72 hours later, drug resistant cells treated with thiostrepton alone showed a 59% cell survival rate, whilst the combination of docetaxel and thiostrepton indicated a synergy, exhibiting a cell survival rate of 34% in this experiment (Figure 6A). Moreover, in drug resistant cells treated with thiostrepton or thiostrepton and docetaxel, the down-regulation of FOXM1 occurred at 48 h and 24 h following treatment respectively (Figure 6B). The shorter time needed for FOXM1 down-regulation in the co-treated cells may reflect the higher levels of cell death happened when both drugs were administered together. Taken together, shown by our research, the resistance of docetaxel is able to be reversed in gastric cancer cells through the inhibition of FOXM1.

## Discussion

In current study, we demonstrated that the level of FOXM1 expression was significantly higher in gastric cancer than in para-cancer tissues and normal gastric cell lines. Although no significant association was found between FOXM1 expression and any clinical pathological features, FOXM1 amplification was identified as an independent prognostic factor in gastric cancer, and its affection is more significant in patients with advanced stage. Moreover, shown to mediate docetaxel resistance in gastric cancers, FOXM1 was revealed to interfere in microtubule polymerization after the treatment of docetaxel in our research. When we attenuated FOXM1 expression with FOXM1 inhibitor thiostrepton, docetaxel resistance in gastric cancers could be reversed, simultaneously with the down-regulation of FOXM1.

Forkhead transcription factor is a new family of transcription factors, which was officially unified in 2000 [31]. In previous studies, FOXM1 was revealed to promote cell cycle by directly activating its downstream genes [19,20]. Furthermore, the overexpressed FOXM1 could simultaneously induce gastric cancer angiogenesis and progression through regulating the level of VEGF (vascular endothelial growth factor) gene expression and correlating with MVD (microvessel density) [26]. These researches may explain why no association was found between FOXM1 expression and clinical pathological parameters, for the clinical features such as tumor size, depth of invasion and lymph node metastasis cannot fully represent the proliferation activity of the tumor cells, whereas FOXM1 mainly reflected the division of cells. This result was agreed with Kaoru Okada’s study, which also detected the expression of FOXM1 in gastric cancer and showed the positive expression did not correlate with any clinic-pathological features [23]. Moreover, overexpression of FOXM1 was previously reported to contribute to the elevated migratory and invasive abilities in oral cavity squamous cell carcinoma [32], indicating that FOXM1 was associated with an aggressive behavior of tumor cells in vitro. These hypotheses were further provided by our results, which showed that FOXM1 is an independent prognostic factor in gastric cancer. Additionally, in order to address the confound influence of other independent prognostic factors, we performed the size, pT and pTNM stratified analysis according to FOXM1 expression levels. As a result, the prognosis was significantly poorer for patients with positive FOXM1 expression when limited to the same tumor size (>5 cm), depth of invasion (T3 and T4) or TNM stage (III-IV), whilst no significant relationship was found between FOXM1 expression and survival duration for patients with stage T1-2, I-II and smaller tumor size. It can be speculated that some other factor may be more significant than FOXM1 in predicting the prognosis at early stage, such as lymph node metastasis [33], whereas in advanced stage, angiogenesis, which is promoted by FOXM1, may influence the growth velocity of tumor more significantly [26]. Based on these results, it is considerately that FOXM1 should be combined with the tumor size, T stage and TNM stage for predicting the prognosis of gastric cancer, and further studies should focus on exploring the mechanism of FOXM1 on promoting the adverse progression of advanced gastric cancers.

In gastric cancer treatment, classical chemotherapy is largely used besides surgery operation, radiotherapy and novel targeted therapy approaches. For instance, docetaxel is commonly used as single-agent or in combination with other drugs like platinum and fluoropyrimidine (DCF regimen) in a neo-adjuvant or advanced stage setting [34,35]. The chemotherapy based on docetaxel may be effective, because docetaxel was reported to lack cross-resistance with other anti-tumor drugs [4]. However, the resistance to docetaxel did occur and FOXM1 was shown to be a critical molecular for that resistance in gastric cancer by our current research, for which elevated levels of FOXM1 was shown to correlate with lower drug susceptibility, whilst the molecular evolution assay of AGS resulted in significantly more resistant cells possessed FOXM1 overexpression. Additionally, Carr et al. have reported that FOXM1 functioned chemoresistance to a microtubule stabilizing anticancer drug, paclitaxel by directly regulating the microtubule destabilizing protein Stathmin and altering dynamics of microtubule in breast cancer [25]. As docetaxel is also an anticancer agent, which binds to β-tubulin and stabilizes microtubules consisting α/β-tubulin dimers, resulting in mitotic failure like paclitaxel, the same mechanism should occur in gastric cancer cells. Accordingly, through tubulin assay analysis, we found that microtubules in FOXM1 overexpressed cell lines fail to polymerize in response to docetaxol treatment, indicating that FOXM1 did prevent docetaxol induced apoptosis by altering the microtubule dynamics in gastric cancer, however, the downstream genes of FOXM1 was not revealed by our research yet. Moreover, after treated cells with FOXM1 inhibitor thiostrepton, the acquired drug resistance was reversed with the down-regulation of FOXM1 expression, demonstrating the inactivation of FOXM1 was essential for reversing docetaxel resistance and targeting FOXM1 could potentially be a better therapeutic strategy for overcoming the resistance to docetaxel. Nevertheless, this inhibitory effect on FOXM1 has not been analyzed for human gastric cancer so far. Therefore, further studies should be focused on the anti-tumor function of FOXM1 inhibitors, especially in docetaxel-resistant gastric cancer.

## Conclusion

In conclusion, our study showed that FOXM1 was an independent prognostic factor for gastric cancer patients. Further, we also showed that FOXM1 is a critical mediator of docetaxel sensitivity and resistance in gastric cancer cells. Therefore, FOXM1 can be a useful marker for predicting patients’ prognosis and monitoring docetaxel response, which might be a new therapeutic target in docetaxel resistant gastric cancer.

## Abbreviations

FOXM1: Forkhead box transcription factor 1; DCF: Chemotherapy regimen of docetaxel cisplatin and 5-fluorouracil; NSCLC: Non-small cell lung cancer; AJCC: The 7th American Joint Committee on Cancer; DMSO: Dimethyl sulfoxide; IHC: Immunohistochemistry; MDR1: Multi-drug resistant protein 1; VEGF: Vascular endothelial growth factor; MVD: Micro vessel density.

## Competing interests

The authors declare that they have no competing interests.

## Authors’ contributions

XL, WQ, and RY designed the experiment. XL, BL, SL and YY performed the experiments. WQ correlated the data and interpreted results. BL performed the statistical analysis. XL drafted manuscript. WQ, RY and SL made critical revision to manuscript. JL was general supervisor of the study, with contribution to the conception and design of the study, as well as data analysis. All authors have read and approved the final manuscript.
